# Olfactomedin 4 (OLFM4) expression is associated with nodal metastases in esophageal adenocarcinoma

**DOI:** 10.1371/journal.pone.0219494

**Published:** 2019-07-08

**Authors:** Lucia Suzuki, Fiebo J. C. ten Kate, Annieke W. Gotink, Hans Stoop, Michail Doukas, Daan Nieboer, Manon C. W. Spaander, Jan J. B. van Lanschot, Bas P. L. van Wijnhoven, Arjun D. Koch, Marco J. Bruno, Leendert H. J. Looijenga, Katharina Biermann

**Affiliations:** 1 Department of Pathology, Erasmus MC University Medical Center Rotterdam, Cancer Institute, Rotterdam, The Netherlands; 2 Department of Gastroenterology and Hepatology, Erasmus MC University Medical Center Rotterdam, Cancer Institute, Rotterdam, The Netherlands; 3 Department of Public Health, Erasmus MC University Medical Center Rotterdam, Cancer Institute, Rotterdam, The Netherlands; 4 Department of Surgery, Erasmus MC University Medical Center Rotterdam, Cancer Institute, Rotterdam, The Netherlands; National Cancer Center, JAPAN

## Abstract

To date no informative biomarkers exist to accurately predict presence of lymph node metastases (LNM) in esophageal adenocarcinoma (EAC). We studied the discriminative value of Olfactomedin 4 (OLFM4), an intestinal stem cell marker, in EAC. Patients who had undergone esophagectomy as single treatment modality for both advanced (pT2-4) and early (pT1b) adenocarcinoma of the esophagus or gastro-esophageal junction were selected for this study from an institutional database (Erasmus MC University Medical Center, Rotterdam, The Netherlands). Surgical resection specimens of 196 advanced and 44 early EAC were examined. OLFM4 expression was studied by immunohistochemistry and categorized as low (<30%) or high (> = 30%) expression. Low OLFM4 was associated with poor differentiation grade in both advanced (60% vs. 34.8%, p = 0.001) and early EAC (39.1% vs. 9.5%, p = 0.023). LNM were present in 161 (82.1%) of advanced and 9 (20.5%) of early EAC respectively. Low OLFM4 was independently associated with the presence of LNM in advanced EAC in multivariable analysis (OR 2.7; 95% CI, 1.16–6.41; p = 0.022), but not in early EAC (OR 2.1; 95% CI, 0.46–9.84; p = 0.338). However, the difference in association with LNM between advanced (OR 2.7; 95% CI, 1.18–6.34; p = 0.019) and early (OR 2.3; 95% CI, 0.47–11.13; p = 0.302) EAC was non-significant (p = 0.844), suggesting that the lack of significance in early EAC is due to the small number of patients in this group. OLFM4 was not of significance for the disease free and overall survival. Overall, low expression of intestinal stem cell marker OLFM4 was associated with the presence of LNM. Our study suggests that OLFM4 could be an informative marker with the potential to improve preoperative assessment in patients with EAC. Further studies are needed to confirm the value of OLFM4 as a biomarker for LNM.

## Introduction

Esophageal cancer is a common cancer with high incidence and mortality rate, with an estimated 456 000 new cases and 400 000 deaths worldwide in 2012 [1], mostly due to diagnosis at advanced incurable stages with limited treatment options. Different histologic types exist, with esophageal squamous cell carcinoma (ESCC) and adenocarcinoma (EAC) being the most frequently encountered types. While ESCC incidences decline, EAC has been one of the fastest rising malignancies in Western countries [2, 3]. Highest incidence rates per 100.0000 person-years for EAC have been observed in the UK (7.2 in men, 2.5 in women) and the Netherlands (7.1 in men and 2.8 in women) [3].

Metastases to the regional lymph nodes is the most important prognostic factor in EAC patients undergoing treatment with curative intent [4–6]. Accurate pretreatment assessment of nodal status is thus important for both advanced and early lesions. In early EAC, patients eligible for endoscopic treatment only (i.e. not followed by surgical resection) should have a minor risk of LNM, because of the inability to perform a lymphadenectomy during endoscopic resection. However, despite all currently available clinical diagnostic modalities (especially EUS, CT and PET) clinical assessment of nodal status is still suboptimal [7–9]. Therefore, a more reliable tool is urgently needed in both advanced and early EAC.

Olfactomedin 4 (OLFM4, formerly known as hGC-1 or GW112) might be an interesting candidate biomarker in this context. It is a secreted glycoprotein, originally identified as a glycoprotein expressed in the olfactory neuroepithelium of bullfrogs [10]. OLFM4 was first cloned from human myeloblasts and is mainly expressed in the gastro-intestinal tract (stomach, small intestine and colon), prostate and bone marrow [11]. In human colon crypts, OLFM4 co-localizes with LGR5+ intestinal stem cells [12]. OLFM4 positive cells are also found in gastric intestinal metaplasia and Barrett’s esophagus (BE), where it is confined to the base of metaplastic glands, in a similar way as in colon crypts, with gradually increased expression during dysplastic progression [13]. OLFM4 is regulated by G-CSF [11], the transcription factor NF-kappaB [14, 15], and the Wnt/β-catenin pathway [16] and can mediate cell adhesion through its interactions with extracellular matrix proteins such as cadherins and lectins [17].

In gastric cancer, low OLFM4 expression is correlated with poor differentiation grade and the presence of LNM, as well as with adverse survival [18–21]. Similarly, decreasing frequencies of expression along with cancer progression have been found in breast, endometrial, prostate and colon carcinoma amongst others [22–25]. Because no data on OLFM4 in EAC are available yet, this study was undertaken to investigate the association between OLFM4 and presence of LNM and prognosis in both advanced and early EAC. We hypothesized low OLFM4 expression in EAC is associated with the presence of LNM and could be a potential biomarker for stratification of patient treatment.

## Materials & methods

### Patients’ selection & study design

Patients who underwent esophagectomy with curative intent for pathologically confirmed pT2-pT4 adenocarcinoma of the esophagus or gastro-esophageal junction between 1995 and 2016 in the Erasmus MC University Medical Center, Rotterdam were selected for this study. Patients were identified from a prospectively collected institutional database. To assure accurate pathological LNM status, patients treated with surgical resection and at least 12 lymph nodes in the resection specimen were included. Patients with concurrent cancer(s) in other organs and/or those dying from surgical complications (survival < 1 month) were excluded as well as patients that received (neo-) adjuvant chemoradiation therapy ((n)CRT). In addition, all patients with early (pT1b) EAC, treated between 1992–2014 at the Erasmus MC, were investigated. These patients were treated by either primary esophagectomy or endoscopic resection followed by esophagectomy because of poor prognostic criteria found in the endoscopic resection specimen. To increase patient numbers in the early EAC group, patients with early EAC and less than 12 pathologically examined lymph nodes, but available follow-up for more than 60 months were also included.

Clinical and pathological data had been prospectively collected, including age at surgery, sex, tumor location and size, surgical technique, resection margin status, differentiation grade, presence (pN-/ pN+) and number (pN0-3) of pathologically confirmed lymph node metastasis and disease free survival (DFS) and overall survival (OS). Resection margin positivity was defined as presence of tumor cells in the (inked) resection margin (definitions according to the College of American Pathologists (CAP)) [26]. Recurrence was defined as either locoregional or distant during follow-up, which was either a clinical diagnosis and sometimes pathologically confirmed. DFS was defined as time between the date of surgery and first occurrence of disease progression. OS was defined as time between surgery and death. Patients lost to follow-up were censored at the time of the last visit to the outpatient clinics. The TNM system according to the UICC seventh edition was used for pathological grading and staging [4]. However, corresponding to the eighth edition, which shows no changes in the definitions of the T, N, and M categories, only carcinomas with their epicentre within the proximal 2 cm of the cardia (Siewert types I and II tumors) were included [27].

### Specimen characteristics

The hematoxylin-eosin stained slides and tissue blocks were retrieved from the archives of the Department of Pathology at the Erasmus MC University Medical Center and re-assessed for tumor staging, grading and additional immunohistochemical staining (IHC) for OLFM4. From the most representative slide with deepest tumor invasion, the FFPE block was selected and 4 μm thick sections were cut from this block. OLFM4 (clone DIE4M, Cell Signalling ref. 14369) staining was performed using an automated immunostainer (BenchMark Ultra, Ventana Medical Systems, Roche, Tuscon, AZ, USA). In brief, deparaffinization according to BenchMark Ultra protocol and antigen retrieval by CC1 antigen retrieval solution (64 min, ref. 950–124, Ventana Medical Systems) were performed. Tissues were incubated with the primary antibody OLFM4 (32 min, dilution 1:400). Detection was performed with UltraView-DAB (ref. 760–500, Ventana Medical Systems) and amplification with Amplification Kit (ref. 760–080 Ventana Medical Systems). Next, the slides were counterstained with hematoxylin (ref: 790–2208, Ventana Medical Systems) and coverslipped. Each slide contained normal colon tissue as a positive control. Furthermore, normal tissue surrounding the tumor was evaluated for its physiological expression of OLFM4 and to assess background staining. OLFM4 expression was scored based on the percentage of tumor cells with cytoplasmic OLFM4 staining. In addition, the H-score based on predominant staining intensity (no / weak/ moderate/ strong staining) was initially scored in a discovery set (n = 57). When present in the same slide (adjacent to tumor) OLFM4 expression was also evaluated in non-dysplastic Barrett’s esophagus (NDBE). Barrett’s esophagus was defined by metaplasia of the pre-existent squamous epithelium into columnar epithelium containing goblet cells[28]. All OLFM4 stained slides were reviewed independently by two investigators (LS and FK), blinded to the clinical and pathological outcome. In case of disagreement, a consensus was reached by review by both investigators. Specifically, 126 out of 240 cases showed a relatively small difference (1–10%) in scoring, of which the numbers were averaged. In 32 cases a difference of more than 10% was found, and a consensus was reached in a consensus meeting.

### Statistical analysis

The optimal cut-off value of OLFM4 expression was based on receiver operating characteristic (ROC) curve analysis in advanced EAC, and corresponding Youden index (S1 Fig). Based on this evaluation, low OLFM4 was defined as < 30% expression, otherwise OLFM4 was considered to be high. The interobserver variation for the assessment of OLFM4 staining between the two observers was calculated using the intraclass correlation coefficient. Strength of agreement was categorized as follows: 0.00–0.20, poor; 0.21–0.40, fair; 0.41–0.60, moderate; 0.61–0.80, good; and 0.81–1.00, excellent.

Required sample size was not calculated a priori as no pilot data on OLFM4 in EAC was available to determine an expected effect size and it was also predetermined by study constraints. Differences between the advanced and early EAC cohorts were analyzed using Student’s *t* test for normal distributions and the Mann–Whitney *U* test for non-normal distributions of continuous variables, and Pearson’s chi-squared (χ^2^) test for categorical variables. Normality of distributions were assessed using the Shapiro-Wilk Test of Normality and by looking at the histogram plot. Correlations between clinicopathological variables and OLFM4 expression were analysed using χ^2^- test or Fisher’s exact test. Multivariable logistic regression was used to calculate independent associated factors for LNM in the resection specimen (pN+). Only variables that were statistically significant in univariable analysis were included in multivariable analysis. To investigate whether the association of OLFM4 was different in advanced and early EAC we performed a logistic regression analysis containing all relevant confounders, OLFM4 status, early or advanced EAC and the interaction between OLFM4 status and early or advanced EAC.

Kaplan Meier curves were used to plot the 5-year DFS and OS by OLFM4 status and the distribution was analyzed using the log-rank test. Uni- and multivariable Cox proportional hazard models were applied to calculate the association between OLFM4 and survival. In multivariable analysis all clinical and pathological factors which proved to be prognostic for survival in univariable analysis were included (p<0.05). Statistical analysis was performed using SPSS-software (version 22, SPSS IBM inc, Armonk, NY, USA). A p-value of <0.05 (two-sided) was considered statistically significant. This study was reported according to the Reporting recommendations for tumor marker prognostic studies (REMARK, S1 Table) [29].

### Ethical approval

This study was approved by the institutional review board (medical ethical committee) from the Erasmus Medical Center (Rotterdam, The Netherlands).

## Results

### Patient characteristics

**Fig 1:**
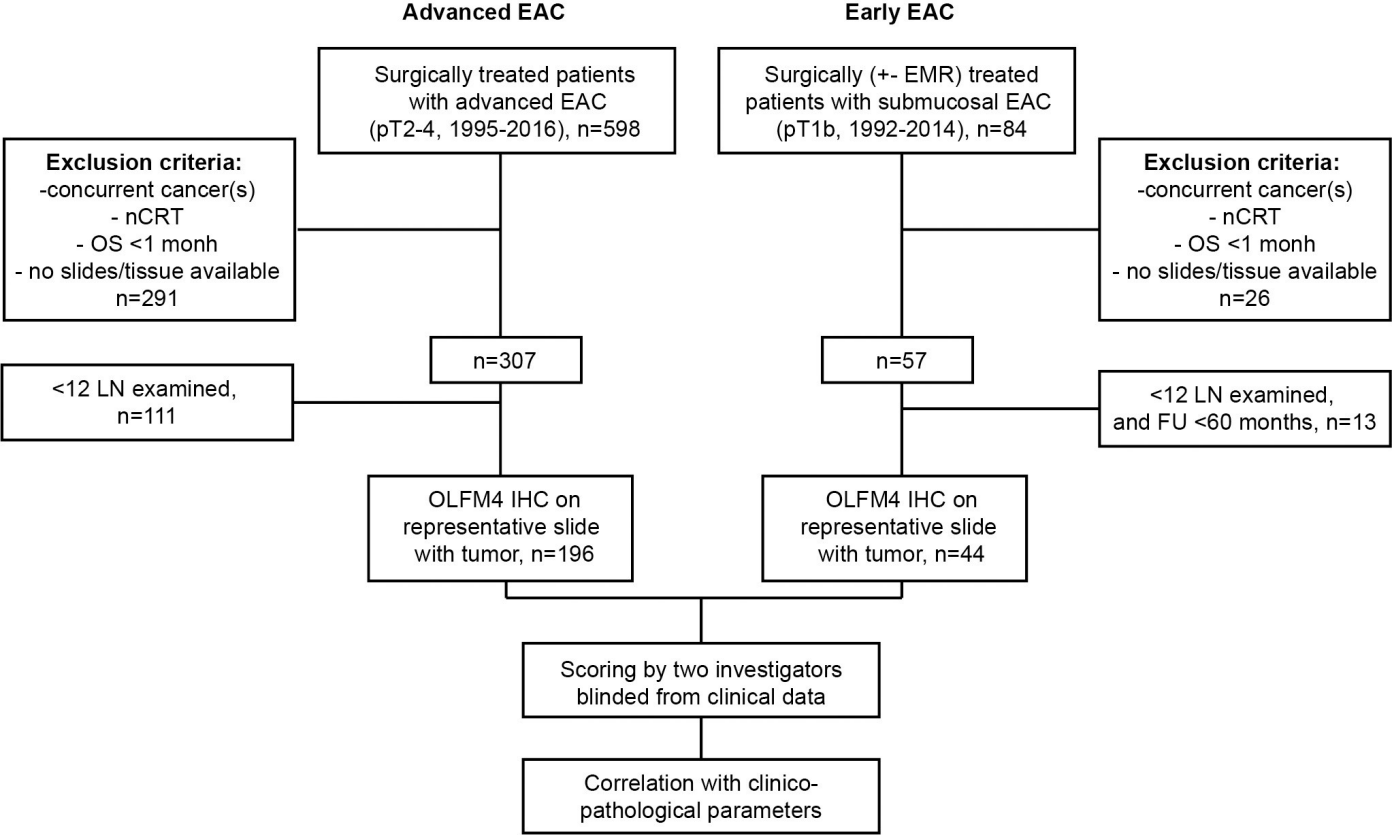
Flow diagram depicting the flow of patients throughout the study. EAC, esophageal adenocarcinoma; nCRT, neo-adjuvant chemo-radiation therapy; OS, overall survival; FU, follow-up, LN, lymph nodes; OLFM4, Olfactomedin 4; IHC, immunohistochemistry.

A diagram depicting the flow of patients throughout the study is shown in Fig 1. Out of 240 EAC patients investigated in this study, 196 had advanced EAC (pT2-4) and 44 early EAC (pT1b). Clinicopathological characteristics are listed in Table 1.

**Table 1:** Patient characteristics*.

		All patients (Advanced + Early),	Advanced EAC (pT2-4),	Early EAC (pT1b),	Advanced vs Early
		n = 240	n = 196	n = 44	p-value*
**Age, years (mean [SD])**		63 (10)	63 (10)	62 (9)	0.445§
**Sex (n[%])**	Male	199 (82.9)	165 (84.2)	34 (77.3)	0.271
	Female	41 (17.1)	31 (15.8)	10 (22.7)	
**Surgery (n[%])**	Transhiatal	150 (62.5)	120 (61.2)	30 (68.2)	**<0.001**
	Transthoracal	69 (28.8)	67 (34.2)	2 (4.5)	
	Total/Partial Gastric	9 (3.7)	8 (4.1)	1 (2.3)	
	Unknown	12 (5.0)	1 (0.5)	11 (25)	
**Siewert classification,¥(n[%])**					
	Type 1	114 (47.5)	80 (40.8)	34 (77.3)	**<0.001**
	Type 2	125 (52.1)	116 (59.2)	9 (20.5)	
**Tumor size, mm▼ (mean [SD])**		46.6 (24.2)	50.6 (23.8)	27.4 (15.8)	**<0.001§**
**Radicality (n[%])**	R0	179 (74.6)	135 (68.9)	44 (100)	**<0.001**
	R1	61 (25.4)	61 (31.1)	0 (0)	
**Grade (n[%])**	Well / Moderate	128 (53.3)	95 (48.5)	33 (75)	**0.001**
	Poor	112 (46.7)	101 (51.5)	11 (25)	
**pT (n[%])**	pT1b	44 (18.3)	0 (0)	44 (100)	**<0.001**
	pT2	25 (10.4)	25 (12.8)	0 (0)	
	pT3	168 (70.0)	168 (85.7)	0 (0)	
	pT4	3 (1.3)	3 (1.5)	0 (0)	
**pN (n[%])**	pN0	70 (29.2)	35 (17.9)	35 (79.5)	**<0.001**
	pN1	49 (20.4)	44 (22.4)	5 (11.4)	
	pN2	54 (22.5)	51 (26.0)	3 (6.8)	
	pN3	67 (27.9)	66 (33.7)	1 (2.3)	
**pN- / pN+ (n[%])**	pN-	70 (29.2)	35 (17.9)	35 (79.5)	**<0.001**
	pN+	170 (70.8)	161(82.1)	9 (20.5)	
**Total LN (median [IQR])**		18 (14–26)	19 (15–27)	14 (8–17)	**<0.001°**
**LNM (median [IQR]**		3 (0–7)	4 (1–8)	0 (0–0)	**<0.001°**
**Recurrence,¶ n[%])**	No	98 (40.8)	66 (33.7)	32 (72.7)	**<0.001**
	Yes	140 (58.3)	130 (66.3)	10 (22.7)	
**Locoregional recurrence,¥ (n[%])**					
	No	163 (67.9)	123 (62.8)	40 (90.9)	**<0.001**
	Yes	76 (31.7)	73 (37.2)	3 (6.8)	
**Distant recurrence,¶ (n[%])**	No	126 (52.5)	94 (48.0)	32 (72.7)	**<0.001**
	Yes	112 (46.7)	102 (52.0)	10 (22.7)	
**pN+ and/ or recurrence,¥ (n[%])**					
	No	49 (20.4)	22 (11.2)	27 (61.4)	**<0.001**
	Yes	190 (79.2)	174 (88.8)	16 (36.4)	
**60 months survival (n[%])**	Alive	79 (32.9)	46 (23.5)	33 (75.0)	**<0.001**
	Deceased	161 (67.1)	150 (76.5)	11 (25.0)	
**Follow-up time, months (median [IQR]**		25 (9–64)	19 (8–48)	38 (47–80)	**<0.001°**
**DFS, months (median [IQR])**		17 (7–60)	13 (6–35)	63 (32–77)	**<0.001°**
**OS, months (median [IQR])**		25 (9–64)	19 (8–48)	64 (47–80)	**<0.001°**
**OLFM4 expression,∞ (n[%])**	Low	153 (63.8)	130 (66.3)	23 (59.0)	0.080
	High	87 (36.2)	66 (33.7)	21 (41.0)	

*P-values were based on Pearson’s chi-squared test, unless indicated otherwise. All statistical tests were two-sided. *SD*, *standard deviation; R1*, *positive; R0*, *negative resection margins; IQR*, *interquartile range*.

**§** P-values were based on Student’s t-test.

° P-values were based on Mann-Whitney test.

¥ One sample (early EAC) had unknown data.

**▼**Eight samples (4 advanced, 4 early EAC) had unknown data.

¶ Two samples (early EAC) had unknown data.

∞ Low OLFM4 < 30% and high OLFM4 ≥30% immunohistochemical expression.

### Pattern of OLFM4 expression

**Fig 2:**
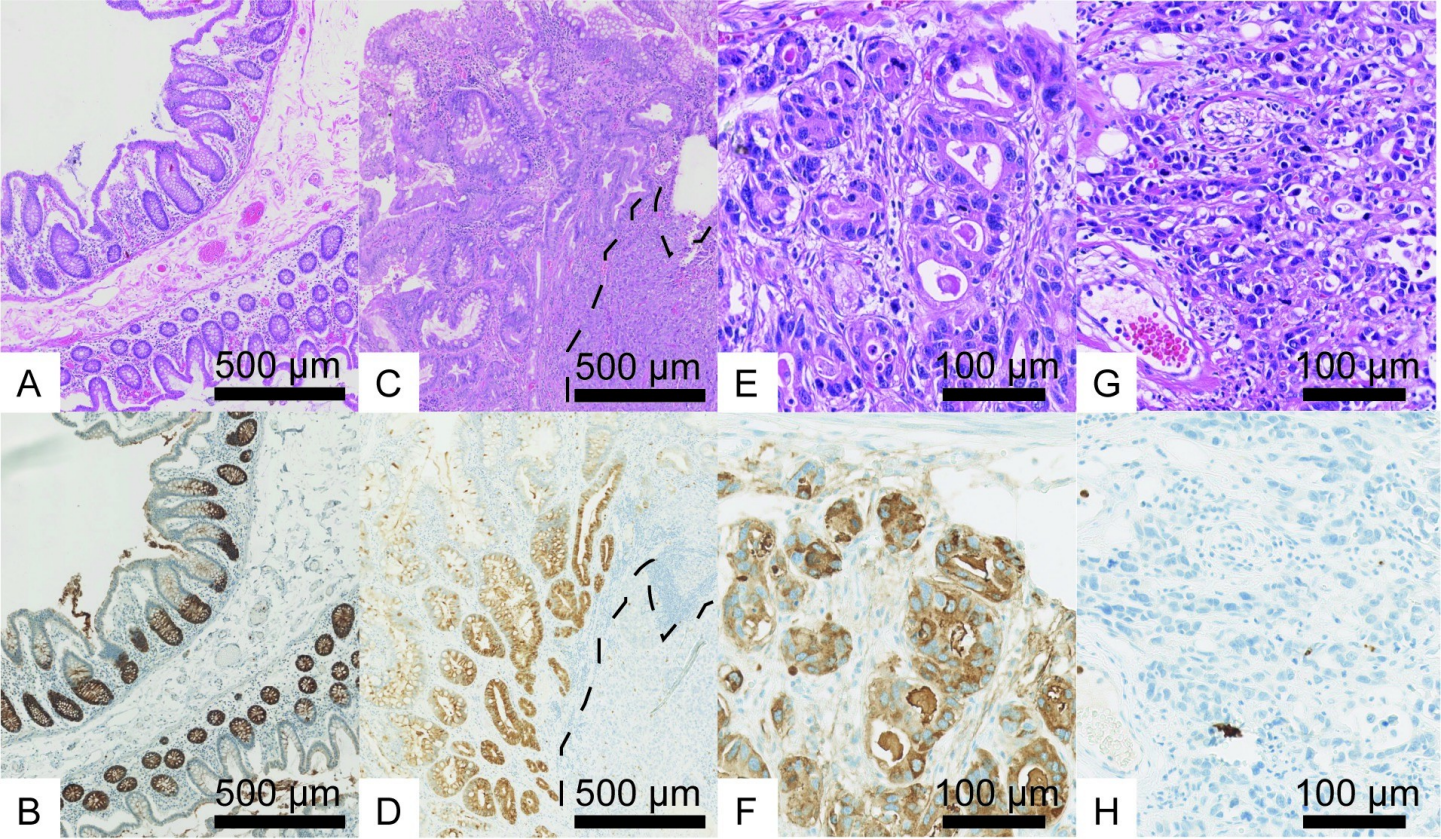
Examples of OLFM4 expression. OLFM4 expression in A, B) normal human colon tissue and C, D) non-dysplastic Barrett’s esophagus overlying OLFM4 negative tumor cells (divided by dotted line). Representative cases of esophageal adenocarcinoma with E, F) high and G, H) low OLFM4 expression (A, C, E, G: hematoxylin- eosin; B, D, F, H: OLFM4).

In total, 240 EAC resection specimens were assessed for OLFM4 expression. The interobserver agreement for OLFM4 assessment was “good” to “excellent” between the two observers with an intraclass correlation co-efficient of 0.871 (95% CI, 0.782–0.918). However, the H-score resulted in a poor interobserver agreement (Cohen’s kappa 0.2) and was disregarded from further analysis. In normal esophageal tissue (without presence of Barrett’s esophagus), OLFM4 expression was absent (S2 Fig). In total, 87 (36.2%) EACs showed high OLFM4 expression and 153 (63.8%) EACs showed low OLFM4 expression (Fig 2). Mostly, expression of OLFM4 was homogeneous, but occasionally, heterogeneous OLFM4 expression was observed, with predominantly high OLFM4 expression towards the lumen and absence of OLFM4 expression towards the invasive front (S3 Fig). Non-dysplastic Barrett’s esophagus (NDBE) showed a similar staining pattern as normal human colon, with cytoplasmic OLFM4 expression in the crypt basis (Fig 2). As in NDBE, OLFM4 expression was noted in the cytoplasm of the EAC cells (Fig 2C).

### OLFM4 expression and clinico-pathological characteristics in advanced and early EAC

In advanced EAC, 78 out of 130 (60%) cases with low OLFM4 expression were poorly differentiated, compared to 23 out of 66 (34.8%) EAC with high expression (p = <0.001, Table 2). A similar association between differentiation grade and OLFM4 expression was found in early EAC (9/23 (39%) vs. 2/21 (10%), p = 0.023). Low OLFM4 expression was also associated with presence of pathologically confirmed LNM at the time of resection in EAC (119/153 (78%) vs 51/87 (59%), p = 0.002). In advanced EAC OLFM4 was associated with LNM (113/130 (87%) vs 48/66 (73%), p = 0.014), but not in early EAC (6/23 (26%) vs 3/21 (14%), p = 0.332).

**Table 2:** Distribution of OLFM4 expression according to clinicopathological characteristics in advanced and early EAC.

		All patients (Advanced + Early), n = 240			Advanced EAC (pT2-4), n = 196			Early EAC (pT1b), n = 44		
		Low n (%)	High n (%)	p-value*	Low n (%)	High n (%)	p-value*	Low n (%)	High n (%)	p-value*
**Age**	<65	81 (52.9)	46 (52.9)	0.992	68 (52.3)	34 (51.5)	0.916	13 (56.5)	12 (57.1)	0.967
	> = 65	72 (47.1)	41 (47.1)		62 (47.7)	32 (48.5)		10 (43.5)	9 (42.9)	
**Sex**	Male	129 (84)	70 (80.5)	0.446	111 (85.4)	54 (81.8)	0.518	18 (78.3)	16 (76.2)	0.870
	Female	24 (15.7)	17 (19.5)		19 (14.6)	12 (18.2)		5 (21.7)	5 (23.8)	
**Surgery**	Transhiatal	93 (60.8)	57 (65.5)	0.467	76 (58.5)	44 (66.7)	0.265	17 (73.9)	13 (61.9)	0.393
	Other	60 (39.2)	30 (34.5)		54 (41.5)	22 (33.3)		6 (26.1)	8 (38.1)	
**Siewert Classification, ¥**,										
	Type 1	75 (49.3)	39 (44.8)	0.501	57 (43.8)	23 (34.8)	0.226	18 (81.8)	16 (76.2)	0.650
	Type 2	77 (50.7)	48 (55.2)		73 (56.2)	43 (65.2)		4 (18.2)	5 (23.8)	
**Tumor Size, ▼**	<5 cm	85 (57.4)	44 (52.4)	0.457	66 (52.0)	28 (43.1)	0.243	19 (90.5)	16 (84.2)	0.550
	> = 5 cm	63 (42.6)	40 (47.6)		61 (48.0)	37 (56.9)		2 (9.5)	3 (15.8)	
**Radicality**	R0	115 (75)	64 (73.6)	0.784	92 (70.8)	43 (65.2)	0.422	23 (100)	21 (100)	NA
	R1	38 (24.8)	23 (26.4)		38 (29.2)	23 (34.8)		0 (0)	0 (0)	
**Grade**	Well/ moderate	66 (43.1)	62 (71.3)	**<0.001**	52 (40.0)	43 (65.2)	**0.001**	14 (60.9)	19 (90.5)	**0.023**
	Poor	87 (56.9)	25 (28.7)		78 (60.0)	23 (34.8)		9 (39.1)	2 (9.5)	
**pT**	pT12	38 (24.8)	31 (35.6)	0.076	15 (11.5)	10 (15.2)	0.474	23 (100)	21 (100)	NA
	pT34	115 (75)	56 (64.4)		115 (88.5)	56 (84.8)		0 (0)	0 (0)	
**pN**	pN0	34 (22.2)	36 (41.4)	**0.008**	17 (13.1)	18 (27.3)	**0.040**	17 (73.9)	18 (85.7)	0.380
	pN1	33 (21.6)	16 (18.4)		29 (22.3)	15 (22.7)		4 (17.4)	1 (4.8)	
	pN2	42 (27.5)	12 (13.8)		40 (30.8)	11 (16.7)		2 (8.7)	1 (4.8)	
	pN3	44 (28.8)	23 (26.4)		44 (33.8)	22 (33.3)		0 (0)	1 (4.8)	
**pN- / pN+**	pN-	34 (22.2)	36 (41.4)	**0.002**	17 (13.1)	18 (27.3)	**0.014**	17 (73.9)	18 (85.7)	0.332
	pN+	119 (78)	51 (58.6)		113 (86.9)	48 (72.7)		6 (26.1)	3 (14.3)	
**Recurrence (loco- regional or distant), ¶**										
	No	54 (35.8)	44 (50.6)	**0.025**	39 (30.0)	27 (40.9)	0.127	15 (71.4)	17 (81.0)	0.469
	Yes	97 (64.2)	43 (49.4)		91 (70.0)	39 (59.1)		6 (28.6)	4 (19.0)	
**pN+ and/ or Recurrence, ¥**										
	No	22 (14.5)	27 (31.0)	**0.002**	10 (7.7)	12 (18.2)	**0.028**	12 (54.5)	15 (71.4)	0.252
	Yes	130 (85.5)	60 (69.0)		120 (92.3)	54 (81.8)		10 (45.5)	6 (28.6)	

*Pearson’s chi-squared test. *NA*, *not applicable*, *because all patients with early EAC had negative resection margins (R0) and were per definition staged pT1*.

¥ One sample (early EAC) had unknown data.

**▼**Eight samples (4 advanced, 4 early EAC) had unknown data

¶ Two samples (early EAC) had unknown data.

To identify the odds ratio (OR) of clinicopathological characteristics for presence of LNM in EAC, uni- and multivariable logistic regression analysis were performed (Table 3). In multivariable analysis, positive resection margin (OR 7.8, 95% CI, 1.70–35.68, p = 0.008), higher pT-stage (pT34, OR 4.0; 95% CI, 1.53–10.29, p = 0.005) and low OLFM4 expression (OR 2.7; 95% CI, 1.16–6.41; p = 0.022) were identified as independent prognostic variables for LNM in advanced EAC. In contrast, no independently prognostic variables were found in early EAC. However, in the combined cohort the interaction test showed no significant difference in strength of the association of OLFM4 with LNM in advanced (OR 2.7.; 95% CI, 1.18–6.34; p = 0.019) and early (OR 2.3; 95% CI, 0.47–11.13; p = 0.302) EAC (p = 0.844, Table 4). In other words, this test shows that there is no reason to assume that the association between OLFM4 and presence of LNM is different between both groups.

**Table 3:** Logistic regression analysis to evaluate the independent association of OLFM4 with LNM (pN+)*.

	All patients (Advanced + Early EAC, n = 240)				Advanced EAC (pT2-4, n = 196)				Early EAC (pT1b, n = 44)	
	Univariable		Multivariable		Univariable		Multivariable		Univariable	
	OR (95% CI)	p-value	OR (95% CI)	p-value	OR (95% CI)	p-value	OR (95% CI)	p-value	OR (95% CI)	p-value
**Age**										
> = 65 (<65 = ref.)	1.1 (0.62–1.89)	0.785			1.0 (0.47–2.02)	0.936			1.1 (0.24–4.66)	0.932
**Sex**										
Male (Female = ref.)	1.3 (0.64–2.71)	0.442			1.1 (0.42–3.00)	0.813			1.0 (0.18–6.02)	0.968
**Surgery**										
Other (Transhiatal = ref.)	1.0 (0.58–1.82)	0.942			0.9 (0.45–1.98)	0.870			0.5 (0.10–3.06)	0.492
**Siewert Classification, ¥**										
Type 2 (Type 1 = ref.)	1.9 (1.10–3.44)	**0.022**	0.9 (0.44–2.01)	0.867	1.0 (0.46–2.02)	0.914			1.1 (0.19–6.52)	0.915
**Tumor Size, ▼**										
> = 5 cm (<5 cm = ref.)	2.5 (1.33–4.53)	**0.004**	1.9 (0.90–4.00)	0.095	1.2 (0.58–2.55)	0.609			2.7 (0.37–19.15)	0.330
**Radicality**											
R1 (R0 = ref.)	18 (4.28–76.37)	**<0.001**	8.3 (1.83–37.76)	**0.006**	9.5 (2.21–41.21)	**0.003**	7.8 (1.70–35.68)	**0.008**		NA
**Grade**										
Poor (Well/ moderate = ref.)	2.7 (1.49–4.87)	**0.001**	1.1 (0.50–2.37)	0.841	2.4 (1.10–5.09)	**0.027**	1.2 (0.48–2.78)	0.751	0.8 (0.14–4.73)	0.829
**pT**										
pT34 (pT12 = ref.)	14 (7.03–26.87)	**<0.001**	7.2 (3.27–15.80)	**<0.001**	5.9 (2.42–14.60)	**<0.001**	4.0 (1.53–10.29)	**0.005**		NA
**OLFM4 expression**										
Low (High = ref.)	2.5 (1.39–4.38)	**0.002**	2.6 (1.22–5.62)	**0.013**	2.5 (1.19–5.25)	**0.016**	2.7 (1.16–6.41)	**0.022**	2.1 (0.46–9.84)	0.338

* Uni- and multivariable logistic regression analysis was performed to investigate the independent association between LNM and clinicopathological characteristics, only variables significant in univariable analysis were included in multivariable analysis. Hence, no multivariable analysis for early esophageal adenocarcinoma (EAC) was performed. *OR*, *odds ratio; CI*, *confidence interval; ref*., *reference; NA = not applicable*, *because all patients with early EAC had negative resection margins (R0) and were per definition staged pT1*.

¥ One sample (early EAC) had unknown data.

**▼**Eight samples (4 advanced, 4 early EAC) had unknown data.

**Table 4:** Multivariable logistic regression analysis to evaluate the association of OLFM4 with LNM (pN+) in all patients (left) with corresponding interaction model (right)*.

		OR	p-value	OR	p-value
**Siewert Classification, ¥**	Type 2 (Type 1 = ref.)	1.0 (0.48–2.11)	0.992	1.0 (0.48–2.11)	0.992
**Tumor Size, ▼**	> = 5 cm (<5 cm = ref.)	1.6 (0.76–3.37)	0.217	1.6 (0.76–3.37)	0.217
**Radicality**	R1 (R0 = ref.)	10.4 (2.32–46.73)	**0.002**	10.5 (2.33–47.20)	**0.002**
**Grade**	Poor (Well/ moderate = ref.)	1.1 (0.52–2.44)	0.762	1.1 (0.52–2.44)	0.767
**pT**	pT234 (pT1 = ref.)	8.2 (3.17–21.12)	**<0.0001**	7.3 (1.75–30.77)	**0.007**
**OLFM4 expression**	Low (High = ref)				
Early EAC, n = 44	pT1			2.3 (0.47–11.13)	0.302
Advanced EAC, n = 196	pT234			2.7 (1.18–6.34)	**0.019**
All patients, n = 240	pT1234	2.6 (1.24–5.62)	**0.012**		
**Interaction§** (Early vs Advanced)				1.2 (0.21–6.91)	0.844

* Only variables significant in univariable analysis were included in multivariable analysis. *OR*, *odds ratio; ref*., *reference; R1*, *positive; R0*, *negative resection margins*

¥ One sample (early EAC) had unknown data.

▼Eight samples (4 advanced, 4 early EAC) had unknown data.

**§** The interaction variable indicates whether there is a difference in association of OLFM4 with LNM between early and advanced EAC. The model with separate effects of OLFM4 for early and advanced EAC did not give a better fit than the model with one effect only.

### OLFM4 expression and prognosis

DFS was significantly better in patients with high OLFM4 expression (for advanced and early EAC cohorts combined, log-rank test, p = 0.024). This was confirmed by univariable COX regression analysis (HR 1.5; 95% CI, 1.05–2.15, p = 0.027). However, this observation did not hold in multivariable analysis (S2 Table). There was no significant difference in OS between EAC with low vs. high OLFM4 expression. Hence, OLFM4 expression was not prognostic for OS (S3 Table). Kaplan-Meier curves for both DFS and OS according to OLFM4 expression are depicted in S4 Fig.

## Discussion

This is the first extensive study on OLFM4, an intestinal stem cell marker, in EAC and shows low OLFM4 expression is associated with positive LNM status. Accurate pretreatment staging of patients with early and advanced EAC is important for optimal treatment selection and survival prediction [4–6]. Previous studies have shown that pretreatment staging is frequently inaccurate in EAC [9, 30–33]. In a recent publication on nCRT-naïve patients with standard pre-operative assessment only 35% of patients were preoperatively diagnosed with a correct T- and N-stage [34]. Particularly in patients with early (pT1) EAC, prevalence of LNM is highly variable and to date unpredictable, while positive LNM status is highly predictive for a poor 5-year survival [30, 35].

The significance of OLFM4 in cancer is still controversial. OLFM4 is able to interact with cell surface proteins and known to facilitate cell-cell adhesion [17, 36]. OLFM4 has been attributed oncogenic properties as it was shown to promote tumor growth by acting as an anti-apoptotic protein and by increasing the mitotic activity of cancer cells [37, 38]. On the other hand, reduced OLFM4 expression was significantly associated with poor prognosis in patients with gastric [20], colorectal [23, 39] and breast carcinoma [24] amongst others. In gastric carcinoma OLFM4 was also associated with metastasis [20, 21].

The present study shows low OLFM4 expression was associated with poorly differentiated EAC, this is in concordance with the literature. In fact, in most cancers, a strong association between low OLFM4 and poor tumor differentiation grade was found, including gastric, colon, ovarian and prostate cancer [17, 18, 21, 22, 27, 28]. These findings suggest tumor suppressive properties of OLFM4 and are in line with results found in various functional studies [16, 40, 41]. For example, in gastric cancer cell lines OLFM4 had an inhibitory effect on cell invasion via regulation of focal adhesion kinase (FAK) signaling [41].

Furthermore, low OLFM4 expression, but not poor tumor differentiation, was independently associated with LNM in advanced EAC in the present study. Because LNM status is critical for the choice of treatment in early EAC, the investigation was extended to early EAC and 44 patients with pT1b tumors were separately analyzed. The overall incidence of LNM in the pT1b group (20.5%) was in line with previous reports [29, 30]. Similar as in advanced EAC, loss of OLFM4 was associated with poor differentiation grade, but no association with LNM status was found. However, the interaction test in the combined cohort showed no significant difference in strength of the association of OLFM4 and LNM between the advanced and early EAC. Therefore, the result in the early EAC might be explained by the small sample size and overall low LNM incidence in this group of patients. Only one previous study studied the role of OLFM4 in early cancer (pT1a and pT1b gastric cancer, n = 105) and concluded that low OLFM4 expression was independently predictive for LNM [21].

Despite the association with LNM, in contrast with results found in other types of cancer, OLFM4 seems to have no effect on clinical outcome. However, there are some important differences between the present study and the aforementioned previous studies on OLFM4 in other cancers. In the present study, only patients with at least 12 lymph nodes resected and identified were included, in order to reduce the percentage of patients with falsely negative pN0 [42]. Although others may have included more cases, these studies were frequently based on patients with various tumor stages and mostly used tissue micro-arrays (TMAs) instead of whole tissue slides. In addition, different methods for scoring OLFM4 IHC were applied making comparison of results difficult [18–23]. Importantly, TMAs may not accurately demonstrate tumor heterogeneity, which was observed in our study occasionally. In addition, whole tissue slides allow for simultaneous analyses of adjacent non-tumorous tissue and Barrett’s esophagus. It would be very interesting to investigate OLFM4 expression in low-grade and high-grade dysplasia. However, in our samples, BE, with or without dysplasia, was present in only a limited number of cases. Therefore, investigation of OLFM4 expression patterns during neoplastic progression would require a separate study design using well defined sample criteria.

There are also some limitations to the present study. Specifically, all patients were from one academic center. Also, patients were treated with surgery alone, while current guidelines recommend nCRT prior to surgery for advanced EAC. However, additional treatment prior to surgery might influence OLFM4 expression and survival, hence it was decided to use a nCRT-naïve patient cohort.

In conclusion, the present study shows that low OLFM4 expression was independently associated with LNM in EAC and hence might prove useful as a new biomarker. Improved prediction of LNM presence could benefit decision making in treatment of EAC patients. This is particularly important in early EAC where overtreatment can be avoided by endoscopic submucosal resection. More research is required to investigate whether OLFM4 is indeed biologically and clinically relevant in both advanced and early EAC.

## Supporting information

S1 Fig Receiver operating characteristics–curve for OLFM4 expression, according to the % of positive tumor cells (cytoplasm), and corresponding Youden index. (DOCX)

S2 Fig OLFM4 expression in normal esophageal tissue. A, B) Normal esophageal tissue is negative for OLFM4. C, D) Magnification of A. Only neutrophils are OLFM4 positive (brown dots indicated by arrows) and can be used as positive internal control (A, B: hematoxylin- eosin; C, D: OLFM4).
(DOCX)

S3 Fig Two examples of cases with heterogeneous OLFM4 expression. In A, B) tumor invading into the muscularis propria and adventitia can be seen. While the tumor in the mucosa, submucosa and muscularis propria is positive for OLFM4, two complete OLFM4 negative clones invading the surrounding fatty tissue can be seen (dotted line). C, D) Magnification of A, B. E, F) A well differentiated tumor with several OLFM4 positive tumor foci towards the lumen (squamous epithelium) and complete absence (below dotted line) of OLFM4 expression in tumor foci towards the invasive front. G, H) Magnification of E, F (A, C, E, G: hematoxylin- eosin; B, D, F, H: OLFM4).
(DOCX)

S4 Fig Kaplan-Meier curves for disease free survival (DFS, left) and overall survival (OS, right) according to OLFM4 expression. DFS and OS of both cohorts (upper two), patients with advanced (middle two) and patients with early (lower two) esophageal adenocarcinoma. Overall, DFS is better in patients with tumors with high OLFM4 expression, although this difference is only significant when both cohorts are combined (p = 0.024, log-rank test). There is no significant difference in OS between EAC with low vs. high OLFM4 expression (log-rank test).
(DOCX)

S1 Table The REMARK checklist. (DOCX)

S2 Table Cox regression analysis to evaluate the risk for recurrence (DFS). Uni- and multivariable Cox regression analysis was performed to investigate the independent association between disease free survival (DFS) and clinicopathological characteristics, only variables significant in univariable analysis were included in multivariable analysis. Hence, no multivariable analysis for early esophageal adenocarcinoma (EAC) was performed. *HR*, *hazard ratio; CI*, *confidence interval; ref*., *reference; NA = not applicable*, *because all patients with early EAC had negative resection margins (R0) and were per definition staged pT1*. ¥ One sample (early EAC) had unknown data. **▼**Eight samples (4 advanced, 4 early EAC) had unknown data.
(DOCX)

S3 Table COX regression analysis for OS. Uni- and multivariable Cox regression analysis was performed to investigate the independent association between overall survival (OS) and clinicopathological characteristics, only variables significant in univariable analysis were included in multivariable analysis. Hence, no multivariable analysis for early esophageal adenocarcinoma (EAC) was performed. *HR*, *hazard ratio; CI*, *confidence interval; ref*., *reference; NA = not applicable*, *because all patients with early EAC had negative resection margins (R0) and were per definition staged pT1*. ¥ One sample (early EAC) had unknown data. **▼**Eight samples (4 advanced, 4 early EAC) had unknown data.
(DOCX)

## Abbreviations

BE: Barrett’s esophagus
CI: confidence interval
DFS: disease free survival
EAC: esophageal adenocarcinoma
ESCC: esophageal squamous cell carcinoma
FFPE: formalin-fixed paraffin-embedded
G-CSF: granulocyte colony stimulating factor
HGD: high grade dysplasia
HR: hazard ratio
IHC: immunohistochemistry
LNM: lymph node metastasis
LGR5: leucin rich repeat containing G protein coupled receptor 5
nCRT: neo-adjuvant chemoradiation therapy
NDBE: non-dysplastic Barrett’s esophagus
OLFM4: Olfactomedin 4
OR: odds ratio
OS: overall survival
pN0: pathologic examination shows no regional lymph node metastasis
pN+: pathologic examination shows 1–2 (pN1), 3–6 (pN2) or > = 7 (pN3) regional lymph node metastasis
pT1a: pT1b, pathologic examination shows tumor invading the lamina propria (pT1a) or submucosa (pT1b)
pT2-4: pathologic examination shows tumor invading muscularis propria (pT2), adventitia (pT3) or adjacent structures (pT4)
ROC: receiver operating characteristics
TMA: tissue micro-array
TNM: tumor node metastasis
UICC: Union for International Cancer Control
χ^2^- test: Pearson’s chi-squared test
