# Supplementary material for: Olfactomedin 4 (OLFM4) expression is associated with nodal metastases in esophageal adenocarcinoma
Source: PLoS One. 2019 Jul 8;14(7):e0219494. doi: 10.1371/journal.pone.0219494 (PMC6613772; doi:10.1371/journal.pone.0219494)
Supplement: S1 Fig — (DOCX) [file pone.0219494.s001.docx]

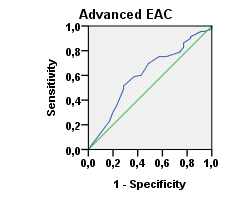


**S1 Fig. Receiver operating characteristics – curve for the semi-quantative OLFM4 expression, according to the % of positive tumor cells (cytoplasm), and corresponding Youden index.**
