# Supplementary material for: Olfactomedin 4 (OLFM4) expression is associated with nodal metastases in esophageal adenocarcinoma
Source: PLoS One. 2019 Jul 8;14(7):e0219494. doi: 10.1371/journal.pone.0219494 (PMC6613772; doi:10.1371/journal.pone.0219494)
Supplement: S2 Fig — A, B) Normal esophageal tissue is negative for OLFM4. C, D) Magnification of A. Only neutrophils are OLFM4 positive (brown dots indicated by arrows) and can be used as positive internal control (A, B: hematoxylin- eosin; C, D: OLFM4). (DOCX) [file pone.0219494.s002.docx]

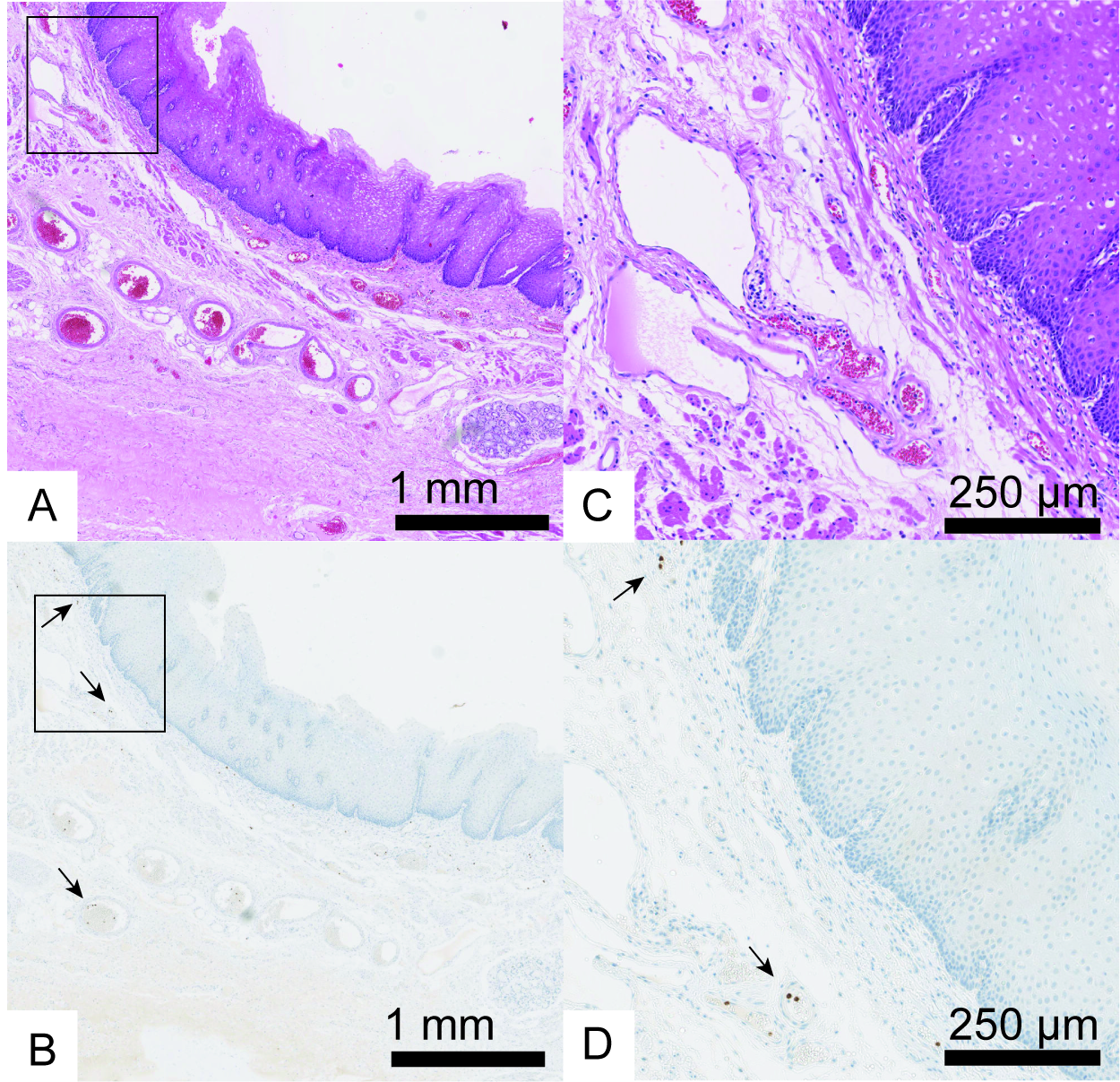


**S2 Fig. OLFM4 expression in normal esophageal tissue.** A, B) Normal esophageal tissue is negative for OLFM4. C, D) Magnification of A, B. Only neutrophils are OLFM4 positive (brown dots indicated by arrows) and can be used as positive internal control (A, B: hematoxylin- eosin; C, D: OLFM4).
