# Supplementary material for: Olfactomedin 4 (OLFM4) expression is associated with nodal metastases in esophageal adenocarcinoma
Source: PLoS One. 2019 Jul 8;14(7):e0219494. doi: 10.1371/journal.pone.0219494 (PMC6613772; doi:10.1371/journal.pone.0219494)
Supplement: S3 Fig — In A, B) tumor invading into the muscularis propria and adventitia can be seen. While the tumor in the mucosa, submucosa and muscularis propria is positive for OLFM4, two complete OLFM4 negative clones invading the surrounding fatty tissue can be seen (dotted line). C, D) Magnification of A, B. E, F) A well differentiated tumor with several OLFM4 positive tumor foci towards the lumen (squamous epithelium) and complete absence (below dotted line) of OLFM4 expression in tumor foci towards the invasive front. G, H) Magnification of E, F (A, C, E, G: hematoxylin- eosin; B, D, F, H: OLFM4). (DOCX) [file pone.0219494.s003.docx]

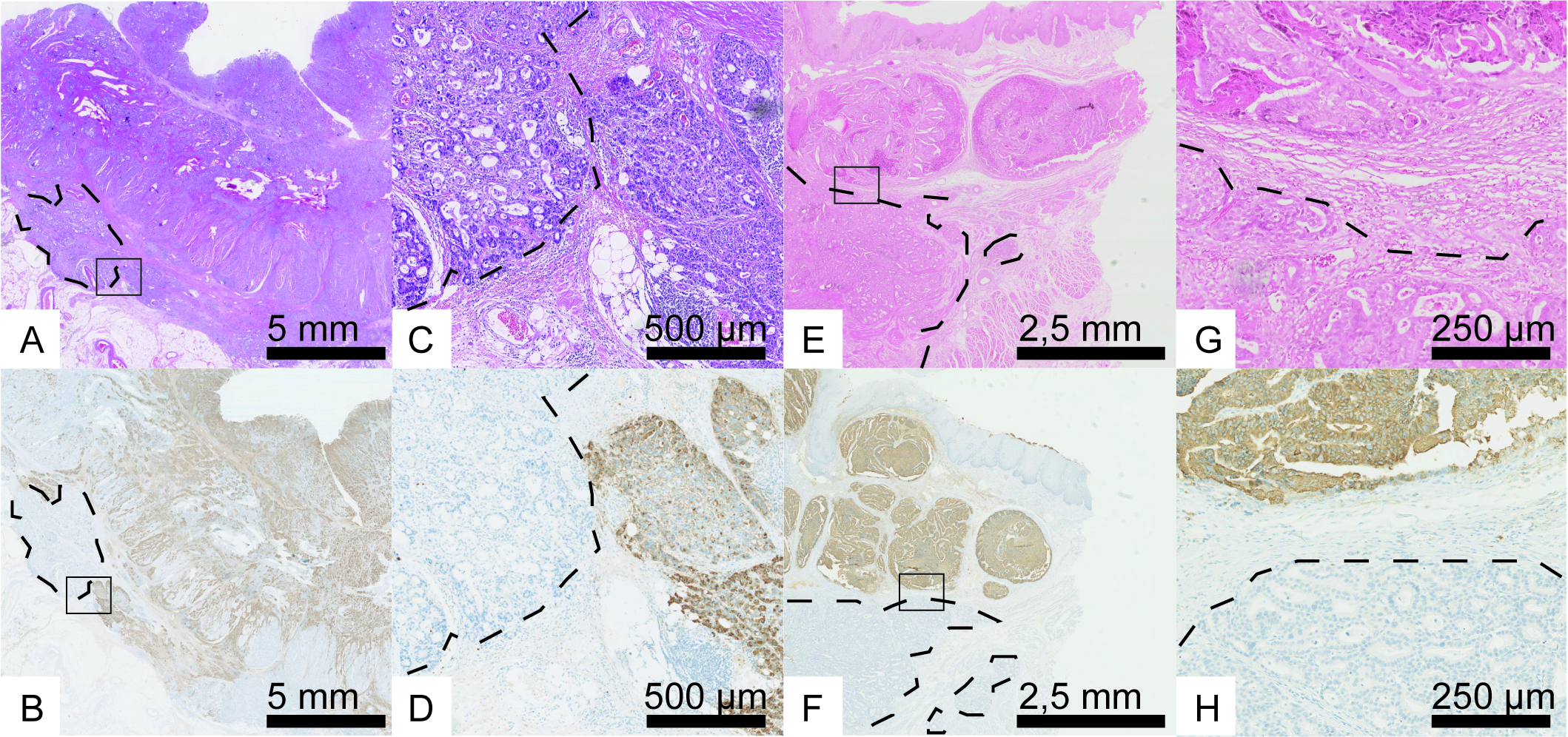


**S3 Fig. Two examples of cases with heterogeneous OLFM4 expression.** In A, B) tumor invading into the muscularis propria and adventitia can be seen. While the tumor in the mucosa, submucosa and muscularis propria is positive for OLFM4, two complete OLFM4 negative clones invading the surrounding fatty tissue can be seen (dotted line). C, D) Magnification of A, B. E, F) A well differentiated tumor with several OLFM4 positive tumor foci towards the lumen (squamous epithelium) and complete absence (below dotted line) of OLFM4 expression in tumor foci towards the invasive front. G, H) Magnification of E, F (A, C, E, G: hematoxylin- eosin; B, D, F, H: OLFM4).
