# Supplementary material for: Olfactomedin 4 (OLFM4) expression is associated with nodal metastases in esophageal adenocarcinoma
Source: PLoS One. 2019 Jul 8;14(7):e0219494. doi: 10.1371/journal.pone.0219494 (PMC6613772; doi:10.1371/journal.pone.0219494)
Supplement: S4 Fig — DFS and OS of both cohorts (upper two), patients with advanced (middle two) and patients with early (lower two) esophageal adenocarcinoma. Overall, DFS is better in patients with tumors with high OLFM4 expression, although this difference is only significant when both cohorts are combined (p = 0.024, log-rank test). There is no significant difference in OS between EAC with low vs. high OLFM4 expression (log-rank test). (DOCX) [file pone.0219494.s004.docx]

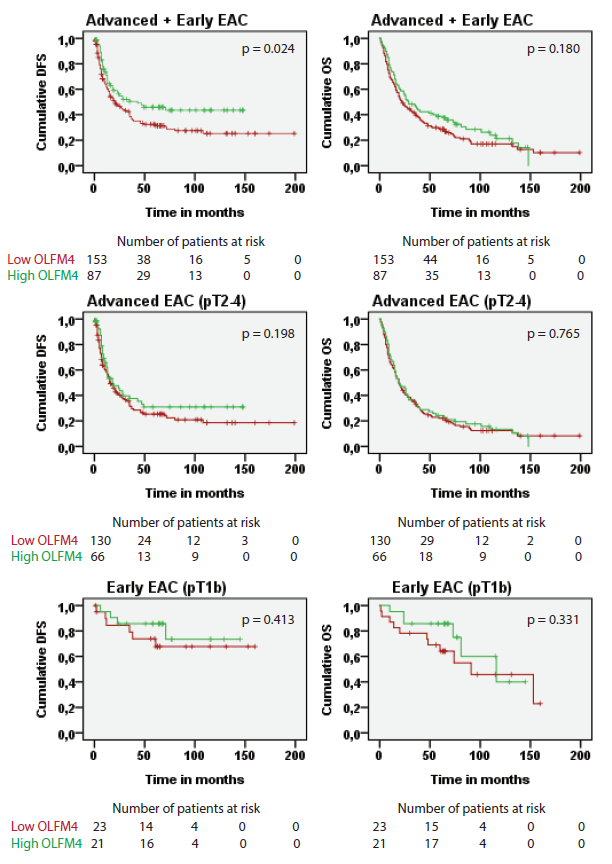


**S4 Fig 4.** **Kaplan-Meier curves for disease free survival (DFS, left) and overall survival (OS, right) according to OLFM4 expression.** DFS and OS of both cohorts (upper two), patients with advanced (middle two) and patients with early (lower two) esophageal adenocarcinoma. Overall, DFS is better in patients with tumors with high OLFM4 expression, although this difference is only significant when both cohorts are combined (p=0.024, log-rank test). There is no significant difference in OS between EAC with low vs. high OLFM4 expression (log-rank test).
