# Supplementary material for: Olfactomedin 4 (OLFM4) expression is associated with nodal metastases in esophageal adenocarcinoma
Source: PLoS One. 2019 Jul 8;14(7):e0219494. doi: 10.1371/journal.pone.0219494 (PMC6613772; doi:10.1371/journal.pone.0219494)
Supplement: S3 Table — Uni- and multivariable Cox regression analysis was performed to investigate the independent association between overall survival (OS) and clinicopathological characteristics, only variables significant in univariable analysis were included in multivariable analysis. Hence, no multivariable analysis for early esophageal adenocarcinoma (EAC) was performed. HR, hazard ratio; CI, confidence interval; ref., reference; NA = not applicable, because all patients with early EAC had negative resection margins (R0) and were per definition staged pT1. ¥ One sample (early EAC) had unknown data. ▼Eight samples (4 advanced, 4 early EAC) had unknown data. (DOCX) [file pone.0219494.s007.docx]

**S3 Table. COX regression analysis for OS.**

|  | **All patients**  **(Advanced + Early EAC, n=240)** | | | | | | **Advanced EAC**  **(pT2-4, n=196)** | | | | | | **Early EAC**  **(pT1b, n=44)** | | |
| --- | --- | --- | --- | --- | --- | --- | --- | --- | --- | --- | --- | --- | --- | --- | --- |
|  | **Univariable** | | | **Multivariable** | | | **Univariable** | | | **Multivariable** | | | **Univariable** | | |
|  | HR  (95% CI) | | p-value | HR  (95% CI) | | p-value | HR  (95% CI) | | p-value | HR  (95% CI) | | p-value | HR  (95% CI) | | p-value |
| **Age** |  |  |  |  |  |  |  |  |  |  |  |  |  |  |  |
| >=65 (<65 = ref.) | 1.3  (1.01- 1.79) | | 0.045 |  |  |  | 1.3  (0.96-1.75) | | 0.097 |  |  |  | 1.9  (0.72-5.00) | | 0.194 |
| **Sex** |  |  |  |  |  |  |  |  |  |  |  |  |  |  |  |
| Male (Female = ref.) | 1.3  (0.86-1.87) | | 0.240 |  |  |  | 1.1  (0.76-1.71) | | 0.526 |  |  |  | 2.3  (0.52-10.17) | | 0.270 |
| **Surgery** |  |  |  |  |  |  |  |  |  |  |  |  |  |  |  |
| Other  (Transhiatal = ref.) | 0.8  (0.58-1.06) | | 0.119 |  |  |  | 0.8  (0.58-1.08) | | 0.141 |  |  |  | 0.5  (0.15- 1.41) | | 0.172 |
| **Siewert Classification, ¥** |  |  |  |  |  |  |  |  |  |  |  |  |  |  |  |
| Type 2  (Type 1 = ref) | 1.1  (0.81-1.45) | | 0.599 |  |  |  | 0.8  (0.59-1.08) | | 0.143 |  |  |  | 0.6  (0.13-2.63) | | 0.490 |
| **Tumor Size, ▼** |  |  |  |  |  |  |  |  |  |  |  |  |  |  |  |
| >= 5 cm  (<5 cm = ref.) | 1.4  (1.02-1.82) | | **0.038** | 1.1  (0.81-1.47) | | 0.558 | 1.0  (0.75-1.38) | | 0.895 |  |  |  | 1.6  (0.43-5.88) | | 0.493 |
| **Radicality** |  |  |  |  |  |  |  |  |  |  |  |  |  |  |  |
| R1 (R0 = ref.) | 2.4  (1.72-3.23) | | **<0.001** | 1.4  (0.98-1.96) | | **0.065** | 1.8  (1.34-2.55) | | **<0.001** | 1.4  (1.01-2.01) | | **0.042** |  |  | NA |
| **Grade** |  |  |  |  |  |  |  |  |  |  |  |  |  |  |  |
| Poor (Well/ moderate = ref.) | 1.9  (1.40-2.51) | | **<0.001** | 1.4  (1.05-1.96) | | **0.023** | 1.4  (1.04-1.92) | | **0.025** | 1.2  (0.90-1.69) | | 0.200 | 4.7  (1.67-13.31) | | **0.003** |
| **pT** |  |  |  |  |  |  |  |  |  |  |  |  |  |  |  |
| pT34  (pT12 = ref.) | 3.1  (2.14-4.45) | | **<0.001** | 1.7  (1.10-2.57) | | **0.016** | 2.0  (1.26-3.29) | | **0.004** | 1.4  (0.87-2.38) | | 0.158 |  |  | NA |
| **pN- vs pN+** |  |  |  |  |  |  |  |  |  |  |  |  |  |  |  |
| pN+  (pN- = ref.) | 3.3  (2.28-4.78) | | **<0.001** | 2.1  (1.39-3.27) | | **0.001** | 2.5  (1.62-3.92) | | **<0.001** | 2.0  (1.28-3.25) | | **0.003** | 2.0  (0.68-5.89) | | 0.209 |
| **OLFM4 expression** |  |  |  |  |  |  |  |  |  |  |  |  |  |  |  |
| Low (High = ref.) | 1.2  (0.91-1.66) | | 0.186 |  |  |  | 1.0  (0.77-1.44) | | 0.769 |  |  |  | 1.6  (0.60-4.53) | | 0.337 |

Uni- and multivariable Cox regression analysis was performed to investigate the independent association between overall survival (OS) and clinicopathological characteristics, only variables significant in univariable analysis were included in multivariable analysis. Hence, no multivariable analysis for early esophageal adenocarcinoma (EAC) was performed. *HR, hazard ratio; CI, confidence interval; ref., reference; NA= not applicable, because all patients with early EAC had negative resection margins (R0) and were per definition staged pT1.*

¥ One sample (early EAC) had unknown data.

**▼**Eight samples (4 advanced, 4 early EAC) had unknown data.
